# Evaluation of optimal dosage combinations of oral midazolam and esketamine for pediatric preoperative sedation: A randomized, double-blind clinical trial

**DOI:** 10.1016/j.clinsp.2026.100921

**Published:** 2026-03-28

**Authors:** Hongfei Xiong, Yingxue Xu, Jing Liu, Guangbo Liu, Yunyun Zhang, Ziwen Wei, Wenwen Liang, Xuemei Zhao, Siyuan Li, Gang Xiao, Rongliang Xue

**Affiliations:** a Anesthesia & Comfort Medical Center, Xi’an International Medical Center Hospital, Xi’an, Shaanxi, China; b Clinical Research Center of Shaanxi Province for Dental and Maxillofacial Diseases, College of Stomatology, Xi’an Jiaotong University, Xi’an, Shaanxi, China; c Department of Anesthesia, Affiliated Hospital of Shandong University of Traditional Chinese Medicine, Jinan, Shandong, China; d The Second Affiliated Hospital of Xi’an Medical College, Xi’an, Shaanxi, China

**Keywords:** Esketamine, Midazolam, Pediatric patients, Oral, Preoperative anxiety

## Abstract

**Objective:**

To evaluate the efficacy and safety of combinations of oral midazolam and esketamine for pediatric preoperative sedation.

**Intervention:**

Children aged 1‒6-years undergoing elective surgery were assigned to Group A (0.62 mg/kg midazolam and 2.05 mg/kg esketamine), Group B (0.41 mg/kg midazolam and 4.1 mg/kg esketamine), and Group C (0.21 mg/kg midazolam and 6.16 mg/kg esketamine). The combination was administered orally before anesthesia induction.

**Outcomes:**

Primary outcome was sedation success, defined as a modified Ramsay sedation score ≥3a, low parent-child separation anxiety, and acceptance of the face mask for inhalation induction. Secondary outcomes included parental satisfaction, occurrence of Adverse Events (AE), and recovery times.

**Results:**

The sedation success rate in Group B was 71.8%, which was significantly higher than Group A (53.5%, *p* < 0.05) but not Group C (68.2%, *p* > 0.05). There was no difference in the sedation success rate between Group A and Group C (*p* > 0.05). The overall incidence rate of AE in Group C was 25.90%, which was significantly higher than that in Group A (2.30%) and Group B (4.70%) (*p* < 0.05). No significant differences in sedation success rates were observed between genders within each group (*p* > 0.05).

**Conclusion:**

The combination of 0.41 mg/kg midazolam and 4.1 mg/kg esketamine provided the most effective sedation with the fewest adverse events in pediatric patients. This regimen may reduce rescue sedation needs and improve perioperative flow.

**Trial registration:**

http://www.chictr.org.cn (ChiCTR2400080633; date of first registration: February 4th, 2024).

## Highlights

• Oral midazolam and esketamine together provide satisfactory sedation in children.
• Oral administration of 0.41 mg/kg midazolam and 4.1 mg/kg esketamine is safe.
• A higher esketamine ratio can cause more adverse events.

## Introduction

Preoperative anxiety is a prevalent and distressing issue among children, particularly in preschoolers, posing significant challenges for both patients and healthcare providers. Research indicates that >50% of children exhibit varying degrees of anxiety responses during anesthesia induction, ranging from mild apprehension to severe distress.^1^ This heightened anxiety can have a detrimental impact on the perioperative experience, leading to delayed recovery, increased analgesic requirements, and the emergence of new behavioral disturbances. In severe cases, preoperative anxiety may even contribute to long-term cognitive impairment.^2^^,^^3^

The physiological manifestations of preoperative anxiety further complicate the anesthesia induction process. The associated surge in stress hormones, including serum cortisol and epinephrine, can lead to hemodynamic instability, posing challenges for achieving a smooth and stable anesthesia induction.^4^ Therefore, selecting the most effective and safe medication for pediatric preoperative sedation is crucial to ensure a positive and uneventful perioperative experience for these vulnerable patients.

Oral midazolam and ketamine have emerged as promising agents for pediatric preoperative sedation due to their rapid onset of action, anxiolytic and sedative properties, and favorable safety profiles. The use of these drugs in combination may offer potential advantages over their individual administration. For instance, combining midazolam and ketamine could enhance the sedative and anxiolytic effects while potentially reducing the required dosage of each drug, thereby minimizing adverse effects.^5^ Furthermore, the synergistic interaction between these two medications may provide a more comprehensive and effective approach to managing preoperative anxiety in children.^6^ To date, the optimal dosage ratio of midazolam and esketamine for combined oral administration remains unexplored. Previous studies have investigated the efficacy of varying dosages of each medication individually, but the synergistic effects of combined administration and the identification of the most effective dosage ratio require further investigation.

This study aims to address this gap in knowledge by systematically evaluating the efficacy and safety of different dosage ratios of oral midazolam and esketamine in reducing preoperative anxiety in children. The findings of this study will provide valuable insights into optimizing the use of this medication combination for pediatric preoperative sedation, thereby improving the perioperative experience and overall well-being of these patients.

## Materials and methods

### Study setting and population

The authors conducted a prospective, double-blinded, randomized, single-center clinical trial. This study was approved by the medical ethics committee, Xi’an International Medical Center Hospital (n° 20,211,205), and written informed consent was obtained by each patient’s legal guardian. The study was registered at www.chictr.org.cn (registration number: ChiCTR2400080633; date of first registration: February 4th, 2024), and conducted at Xi’an International Medical Center Hospital. The Good Clinical Practice guidelines and the guidelines of the Helsinki Declaration were followed in the study. Each patient in this study had written informed consent obtained from his or her legal guardian.

Patients aged 1- to 6-years with a Body Mass Index (BMI) of 13 to 20 kg/m^2^ (calculated according to the growth curves of Chinese children aged 1- to 6-years and converted into age and gender-specific percentiles) and classified as American Society of Anesthesiologists (ASA) I‒II, who were scheduled for elective surgery under general anesthesia, were eligible for recruitment. Exclusion criteria included patients with endocrine system diseases, severe respiratory diseases (such as obstructive sleep apnea syndrome, acute respiratory infections, uncontrolled asthma, active hemoptysis, or pulmonary hypertension), heart disease, mental diseases (including autism, attention deficit hyperactivity disorder, or schizophrenia), abnormal brain development or cognitive dysfunction, coma, brain injury or neurosurgery, abnormal liver function (aspartate aminotransferase and/or alanine transaminase levels ≥2.5 × Upper Limit of Normal (ULN), total bilirubin levels ≥1.5 × ULN), abnormal renal function (urea nitrogen levels ≥1.5 × ULN, serum creatinine levels >ULN), patients with known allergy to benzodiazepines, opiates, propofol, esketamine or other drugs used in the study, used sedative drugs within 48 h before surgery, premature infants, patients who may have ingested alcohol or narcotics for various reasons, including medication and family influence, patients with any other diseases that could potentially interfere with the study results, or incomplete Case Report Forms (CRFs).

### Randomization and blinding

A biostatistician who is unaware of treatments and patient follow-up, generated the random numbers using the SAS software (SAS Institute, USA) in a ratio of 1:1:1, assigning Group A, Group B and Group C. The randomization sequence was stored online until the end of the study (https://pan.baidu.com) in a secure, password-protected format accessible only to the biostatistician via a unique login credential. As new participants consented to join the study, the responsible anesthesiologist would contact the biostatistician, who would assign the participant to either Group A, B, or C based on the next unused random number from the online sequence. This allocation was marked as used in red.

The biostatistician was aware of the randomization sequence and did not participate in the conduct of the study or patient follow-up. To further ensure allocation concealment and blinding integrity, the anesthesia process for each patient was managed by two distinct anesthesiologists with strictly segregated roles. The responsible anesthesiologist was aware of the patient allocation; however, he or she was not involved in the patient assignment, follow-up, outcome assessment, anesthesia record documentation and data analysis. This anesthesiologist's responsibilities were confined exclusively to preparing the study drugs. To maintain allocation concealment, the anesthesiologist preparing the study drugs had no access to outcome data and was explicitly excluded from all patient follow-up and assessment activities, including post-operative recovery monitoring in the Post-Anesthesia Care Unit (PACU), communication with patients or families, and any interactions that could inadvertently influence blinding. All such activities, including anesthesia record completion, post-operative follow-up, and outcome evaluations (e.g., sedation scores, adverse event logging), were delegated to a separate, blinded anesthesiologist who had no knowledge of group allocations and operated independently of the drug preparation process. This role separation minimized performance bias by preventing knowledge of allocations from influencing clinical assessments or patient care. Furthermore, the anesthesiologist responsible for preparation did not interact with patients, parents, or independent assessors at any stage, and all communications regarding allocations were logged in a secure audit trail independent of the clinical workflow. The study drugs were prepared by the responsible anesthesiologist, diluted to the same volume with glucose without labeling. Throughout the study, all study personnel, patients, parents, and everyone except the responsible anesthesiologist remained blind to the patients' allocations.

In an emergency, each center's principal investigator can request the treatment allocation unmasking. The case should be documented and analyzed to evaluate any association with the treatment.

### Intervention and sedation/anesthesia protocol

To explore the optimal combination, the authors designed three dosage ratios based on these Effective Dose 95% (ED95) values: Group A received 75% of the ED95 for midazolam (0.62 mg/kg) and 25% of the ED95 for esketamine (2.05 mg/kg); Group B received 50% of the ED95 for both drugs (0.41 mg/kg midazolam and 4.1 mg/kg esketamine); and Group C received 25% of the ED95 for midazolam (0.21 mg/kg) and 75% of the ED95 for esketamine (6.16 mg/kg). These ratios were chosen to systematically evaluate the synergistic effects of varying proportions of midazolam and esketamine while maintaining a consistent total effective dose relative to their ED95 values. The ED95 for oral midazolam (0.8254 mg/kg) was derived from the authors’ previously published research in a similar pediatric population.^7^ The ED95 for oral esketamine (8.2125 mg/kg) was determined from an unpublished single-center, dose-escalation clinical study conducted at Xi’an International Medical Center Hospital involving 62 pediatric patients aged 1‒6 years with ASA I‒II status undergoing elective surgery (Chinese Clinical Trial Registry: ChiCTR2400082312), matching the current study's demographic and clinical profile. This study employed a modified Dixon’s up-and-down method to estimate the effective dose, with sedation success defined as achieving a modified Ramsay sedation score ≥3a upon separation from parents, a Patient's Separation Anxiety Score (PSAS score) ≤2 points, and Mask Acceptance Score (MAS) ≤2 points upon sevoflurane inhalational anesthesia induction. This study has been submitted for peer review but has not yet been formally published, and the value was internally validated through dose-response modeling, matching the current study's demographic and clinical profile.

Prior to anesthesia, patients were instructed to fast from clear liquids for 2 h and from solid food for 6 h. Upon arrival at the pre-operation holding area, accompanied by parents, patients' vital signs including pulse oximetry (SpO_2_), were monitored as a standard procedure.

A mixture of 2.05 mg/kg esketamine injection (Hengrui Pharma, China) with 0.62 mg/kg midazolam oral solution (Humanwell Pharma, China) was administered orally to the patients from Group A by the patients' parents. For patients from Group B, 4.1 mg/kg esketamine injection with 0.41 mg/kg midazolam oral solution was administered orally. For patients from Group C, 6.16 mg/kg esketamine injection with 0.21 mg/kg midazolam oral solution was administered orally.

After oral administration, serial evaluations were performed by a single independent research personnel. Briefly, sedation level was evaluated every 5 min using the modified Ramsay sedation scale.^8^ Once the modified Ramsay sedation score reached 3a, an attempt was made to separate the patient from their parents, and the PSAS score was recorded.^8^ To ensure inter-observer reliability, all assessments were conducted exclusively by this trained independent assessor, who underwent pre-study calibration training using video-recorded examples of pediatric sedation scenarios, achieving a Cohen's kappa coefficient of >0.8 for inter-rater agreement during mock evaluations with a second observer. Standardized scoring protocols from validated references[8] were adhered to, minimizing subjectivity in mask acceptance and separation anxiety ratings. If the PSAS score was >2, patients continued to wait for up to 30 min for the midazolam to take effect. If the waiting time exceeded 30 min, the separation was considered a failure, and esketamine intramuscular sedation rescue was performed. A single rescue dose of 2 mg/kg was administered in such cases. If the PSAS score was ≤2 points, patients were transferred to the operating room, where routine monitoring was established, including Electrocardiogram (ECG), SpO_2_, Heart Rate (HR), Noninvasive Blood Pressure (NBP), and Bispectral Index (BIS). Inhalation induction with 8% sevoflurane (Maruishi Pharmaceutical, Japan) was conducted, and the acceptance of the face mask by the patient was evaluated by the same independent observer using a 1 to 4 points MAS. 1 point: No fear of the mask and easy acceptance; 2 points: A slight fear of the mask, with the patient being easy to comfort; 3 points: Moderate fear of the mask, with the patient being difficult to calm; 4 points: Fear of the mask, with the patient crying or struggling,^8^ Intravenous puncture was performed when the end-expiratory sevoflurane concentration reached 2 Minimum Alveolar Concentration (MAC). Anesthesia was induced and maintained using the following methods: 3 mg/kg propofol (AstraZeneca, UK), 0.2 mg/kg cisatracurium (Xianju Pharma, China) and 0.4 μg/kg sufentanil (Humanwell Healthcare, China). During surgery, a continuous infusion of 6‒10 mg/kg.h propofol and 0.1‒0.3 μg/kg.min remifentanil (Humanwell Healthcare, China) was adjusted to maintain the BIS between 45‒60 and to keep blood pressure and heart rate within 20% of the baseline. Cisatracurium was administered at a maintenance infusion rate of 2 µg/kg/min during the surgery. Cisatracurium infusion was terminated 30 min before the end of surgery, and 0.5 mg/kg ketorolac tromethamine (Hualu Pharma, China) 0.1 mg/kg dexamethasone (Xinhua Pharma, China), and 0.1 mg/kg tropisetron (Qilu Pharma, China) were administered intravenously. After surgery, all patients were transferred into PACU. Extubation was performed once the patients had fully recovered spontaneous ventilation, and their SpO_2_ was 99%‒100%.

### Outcomes

#### Primary outcome

The primary outcome was the sedation success rate for preoperative anxiety alleviation in children. Sedation success was defined as achieving a modified Ramsay sedation score ≥3a upon separation from parents, a PSAS score ≤2 points, and MAS ≤2 points upon sevoflurane inhalational anesthesia induction, with all components assessed by a single trained independent observer to ensure consistency, as detailed in section 2.3. Inter-observer reliability was confirmed through pre-trial calibration (Cohen's kappa >0.8), reducing variability in subjective elements such as mask acceptance.

#### Secondary outcomes

The secondary outcomes included the patient behavioral score while taking drugs,^9^ the need for sedation rescue, time taken for successful sedation separation (the time from administering the medication to successfully separating the child from the parents, with a modified Ramsay sedation score of at least 3a achieved at the time of separation), parental satisfaction with the separation process, and the occurrence of adverse events during sedation including hypoxemia (SpO_2_ levels < 95%), respiratory depression (SpO_2_ ≤90%, lasting 10 s), nausea, vomiting, and agitation. Additionally, adverse events during the recovery period were also recorded. Adverse events were classified by severity (mild: transient symptoms requiring no intervention; moderate: symptoms requiring minor intervention but resolving without sequelae; severe: symptoms requiring significant intervention or resulting in prolonged effects) and causality (drug-related: definite or probable attribution to study drugs based on temporal association, dechallenge/rechallenge if applicable, and exclusion of alternatives; procedural/other: unrelated to drugs, e.g., due to surgery or anxiety), following standard pharmacovigilance guidelines. The patient's behavioral score was rated on a scale of 1 to 4, with a score of 1 indicating calm and cooperative behavior, 2 indicating anxious but reassurable behavior, 3 indicating anxious and not reassurable behavior, and 4 indicating crying or resisting behavior. Parental satisfaction was measured on a scale of 1 to 5, with a score of 1 indicating very poor satisfaction, 2 indicating poor satisfaction, 3 indicating moderate satisfaction, 4 indicating good satisfaction, and 5 indicating excellent satisfaction.

### Sample size

A pilot study informed the sample size calculation, assuming expected sedation success rates of 55% in the lower-dose midazolam group (analogous to Group A) and 75% in the balanced-dose group (analogous to Group B), representing a 20% difference in the primary outcome (sedation success rate), with an assumed standard deviation of 0.49 (derived from binomial variance √[p(1-p)] using a conservative *p* = 0.5 for maximum variance). Based on a two-tailed alpha of 0.05 and a power of 0.8 (beta = 1 ‒ power = 0.2), the required sample size was 82 patients per group (246 total) for the primary pairwise comparison (Group A vs. Group B), calculated using online sample size calculation software and treating success rates as continuous outcomes (proportions scaled to 0‒1) for approximation in a three-group design using online sample size calculation software. (https://www.cnstat.org/samplesize/mean). Anticipating potential dropouts, the authors increased the initial enrollment to 276 participants.

### Statistical analysis

GraphPad Prism 6.0 software (GraphPad Software Inc., USA) was used to perform the statistical analysis. Both Per-Protocol (PP) and Intention-To-Treat (ITT) analyses were conducted for robustness, with the PP set comprising subjects randomly assigned without major protocol violations (e.g., entry criteria breaches, incorrect treatments, or excluded medications). The ITT set included all randomized patients, with dropouts (*n* = 7, primarily due to refusal of medication) imputed using last observation carried forward for categorical outcomes (e.g., sedation success) and mean imputation for continuous variables (e.g., separation time). The Gaussian distribution was tested using D'Agostino & Pearson analysis. Data was displayed as mean ± Standard Deviation (SD) or the median value (1st‒3rd quartiles). An ordinary one-way ANOVA was used to analyze the numeric variables. χ^2^ test or Fisher's exact probability method was used to analyze the categorical variables. A significant difference was defined as a value of *p* < 0.05. Sensitivity analyses confirmed that ITT results were consistent with PP findings.

## Results

### Demographic and clinical characteristics of the patients

**Fig. 1:**
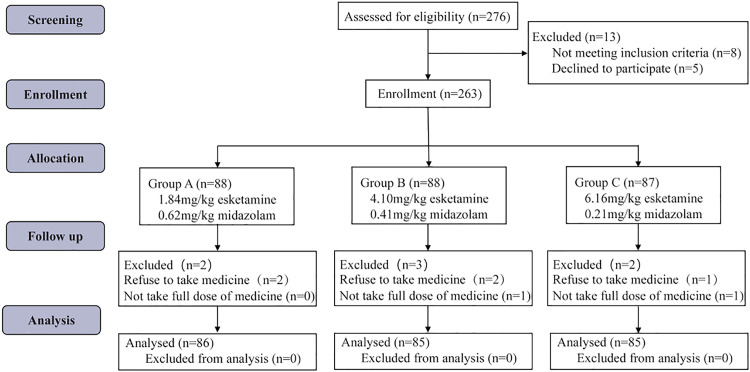
Study flow diagram. Flow of participants through the trial, from screening (*n* = 276) to randomization (*n* = 263) and final per-protocol analysis (*n* = 256), with reasons for exclusions and dropouts noted. Intention-to-treat analysis included all randomized patients (*n* = 263).

A total of 276 patients were screened between March 2024 to August 2024 for the study. Of these, 5 patients were excluded due to their parents' unwillingness to participate, and 8 patients did not meet the inclusion criteria. The remaining 263 patients were enrolled in the study, and randomly allocated into Group A (*n* = 88), Group B (*n* = 88), and Group C (*n* = 87). However, 7 patients dropped out because these children refused to take medication or did not complete the full prescribed dose. Throughout the study period, there was no loss of follow-up among participants. Ultimately, data from a total of 256 patients from Group A (*n* = 86), Group B (*n* = 85), and Group C (*n* = 85) were included in the final per-protocol analysis (Fig. 1). The demographic data of these patients are shown in Table 1. The intention-to-treat analysis included all 263 randomized patients, yielding similar demographic balances across groups (*p* > 0.05). The authors did not find any significant difference in the demographic data between the three groups (*p* > 0.05).

**Table 1:** Comparison of demographic data between groups.

Demographic data	Group A (*n* = 86)	Group B (*n* = 85)	Group C (*n* = 85)	**p**
Male/Female (n)	48/38	49/36	56/29	0.372
Age [median (IQR), year]	3 (2, 4)	3 (1, 4)	3 (2, 4.5)	0.485
BMI (mean±SD, kg/m^2^)	16.11±2.16	16.39±2.07	16.28±2.20	0.700
ASA (I/II)	64/22	65/21	69/16	0.557
Duration of surgery [median (IQR), min]	45.5 (29.75, 70)	38.5 (24.75, 90.25)	35 (25.5, 63.5)	0.271
Intraoperative remifentanil consumption [median (IQR), μg.kg^-1^]	9.2 (5.9, 14.2)	7.6 (4.9, 18.2)	7 (5.1, 12.7)	0.21
Intraoperative propofol consumption [median (IQR), mg.kg^-1^]	6.1 (3.9, 9.5)	5.1 (3.3, 12.1)	4.7 (3.5, 8.5)	0.253

Data are presented as mean ± SD or medium [IQR] for continuous variables (age, weight, BMI) or number (percentage) for categorical variables (gender, ASA status). Statistical analyses: one-way ANOVA for continuous variables; χ² test for categorical variables. No significant differences between groups (all *p* > 0.05).

### Primary outcome

The sedation success rate for preoperative anxiety alleviation in Group A, B and C was 53.50%, 71.80%, and 68.20%, respectively. Group B demonstrated a higher sedation success rate when compared with Group A (*p* < 0.05) but not Group C (*p* > 0.05). The authors did not find a significant difference in the sedation success rate between Group C and Group A (*p* > 0.05). Gender-based analysis revealed no significant differences in sedation success rates between males and females within each group: Group A (males: 54.2%, females: 52.6%, *p* = 0.88), Group B (males: 71.4%, females: 72.2%, *p* = 0.93), and Group C (males: 67.9%, females: 69.0%, *p* = 0.92). The ITT analysis produced comparable results (Group A: 52.3%, B: 70.5%, C: 66.7%; p 〈 0.05 for B vs. A, p 〉 0.05 for other comparisons), confirming the robustness of the per-protocol findings.

### Secondary outcomes

ITT analyses for secondary outcomes (e.g., rescue rates: A 47.7%, B 28.2%, C 31.0%; separation times: medians unchanged) mirrored the per-protocol results, with no substantive differences. Successful sedation separation was defined as the time elapsed from medication administration to successful separation from parents, achieved when the modified Ramsay sedation score reached at least 3a. A total of 46 children in Group A, 61 children in Group B, and 58 children in Group C were successfully separated. The median separation time for all three groups was 15 min. There was no significant difference in separation time between the groups (*p* > 0.05, Table 2).

**Table 2:** Comparison of sedation levels between groups.

	Group A (*n* = 86)	Group B (*n* = 85)	Group C (*n* = 85)	p
**Separation time [median (IQR), min]**	15 (10, 20)	15 (12.5, 20)	15 (10, 20)	0.455
**Sedation levels, n (%)**				
Minimal sedation rate	53 (61.63%)	49 (57.65%)	39 (45.88%)	0.099
Moderate sedation rate	29 (33.72%)	26 (30.59%)	20 (23.53%)	0.325
Deep sedation rate	2 (2.33%)	10 (11.76%)^a^	26 (30.59%)^ab^	<0.01
**Patient behavioral score [median (IQR)]**	1 (1, 2)	1 (1, 4)	1 (1, 2)	0.117
**Parental satisfaction scale [median (IQR)]**	4 (4, 5)	5 (4, 5)^a^	5 (4, 5)^a^	<0.01

Data for separation time, patient behavioral score and parental satisfaction scale are presented as median [IQR] minutes; sedation levels as number (percentage). Statistical analyses: Kruskal-Wallis test for non-normal continuous variables (separation time); χ^2^ test for categorical variables (sedation levels, behavioral scores, satisfaction). No significant differences in separation time, mild/moderate sedation rates, or behavioral scores (all *p* > 0.05); deep sedation rates: *p* < 0.01 (Group C vs. A and B), *p* = 0.016 (Group B vs. A)^a,b^; parental satisfaction: *p* = 0.003 (Group B vs. A), *p* < 0.01 (Group C vs. A), *p* = 1.0 (Group B vs. C).

The rescue sedation rates in Groups A, B, and C were 46.5% (40 of 86), 28.2% (24 of 85), and 31.8% (27 of 85), respectively. Group B exhibited a significantly lower rescue sedation rate than Group A (*p* < 0.05) but not Group C (*p* > 0.05). There was no significant difference in rescue sedation rates between Group A and Group C (*p* > 0.05).

The authors also compared patient sedation levels at the time of separation from parents. As shown in Table 2, Group C exhibited a significantly higher rate of deep sedation than Groups B and A (30.59% vs. 11.76% and 2.33%, respectively, *p* < 0.01). Additionally, Group B demonstrated a higher rate of deep sedation than Group A (*p* = 0.016). For moderate sedation, groups A, B, and C had rates of 33.72%, 30.59%, and 23.53%, respectively, with no significant differences (*p* > 0.05). Similarly, there were no significant differences in mild sedation rates among the groups (Group A: 61.63%, Group B: 57.65%, Group C: 45.88%, *p* > 0.05).

There were no significant differences in patient behavioral scores among the three groups (*p* > 0.05, Table 2). However, parents of patients in Groups B and C reported significantly higher satisfaction with the sedation procedure compared to parents in Group A (*p* = 0.003 and *p* < 0.01, respectively). There was no significant difference in parental satisfaction between Groups B and C (*p* = 1, Table 2).

After surgery, the median of consciousness recovery time in Groups A, B and C was 30 (18, 43), 26 (13.5, 41) and 28 (11, 44) min, respectively. There was no significant difference between the three groups (*p* > 0.05, Table 3). Gender-based analysis of adverse event rates showed no significant differences between males and females within each group: Group A (males: 2.1%, females: 2.6%, *p* = 0.87), Group B (males: 4.1%, females: 5.6%, *p* = 0.71), and Group C (males: 25.0%, females: 27.6%, *p* = 0.78).

**Table 3:** Comparison of anesthesia recovery and adverse event between groups.

	Group A (*n* = 86)	Group B (*n* = 85)	Group C (*n* = 85)	p
**Consciousness recovery time (min)**	30 (18, 43)	26 (13.5, 41)	28 (11, 44)	0.403
**Overall adverse event, n (%)**	2 (2.30)	4 (4.70)	22 (25.90)^ab^	<0.01
**Adverse events during sedation**				
Hypoxemia (SpO_2_ < 95%)	0^c^	0^c^	3	‒
Assisted respiration	0	0	0	‒
Nausea & vomiting and increased secretion	0	1^a^	10^ab^	‒
Agitation	0	0	0	‒
**Adverse events in PACU**				
Nausea, vomiting & increased secretion	0^c^	0^c^	8	‒
Minimal sedation	0^b^	1	0^b^	‒
Anxiety	2^c^	2^c^	0	‒
Agitation	0^c^	0^c^	1	‒

Recovery times are presented as median [IQR] minutes; adverse event rates as number (percentage). Statistical analyses: Kruskal-Wallis test for recovery times (no significant differences, *p* > 0.05); χ^2^ test for adverse event rates. Overall adverse event rates: *p* < 0.01 (Group C vs. A and B)^a,b^; no difference between Groups A and B (*p* = 0.667).

As shown in Table 3, the overall adverse event rate after drug administration and in PACU for Groups A, B and C was 2.30%, 4.70% and 25.90%, respectively. Patients in group C experienced much higher adverse events when compared with Group A (*p* < 0.01) and B (*p* < 0.01). There was no significant difference between Group A and Group B (*p* = 0.667). The major adverse events in Group C were hypoxemia (SpO_2_ levels < 95%), nausea & vomiting and increased secretion. However, the major adverse event in Group A and Group B was anxiety in PACU. Detailed classification by severity and causality is presented in Table 4. In Group C, a significantly higher overall incidence of AEs was observed (41.2%, *p* < 0.01 vs. Groups A and B), predominantly classified as moderate and drug-related. Specifically, 14.1% of patients in Group C experienced nausea/vomiting, and 10.6% had increased secretions, both considered drug-related. Hypoxemia (SpO_2_ < 95%) occurred in 3.5% of patients in Group C, all of whom recovered promptly with supplemental oxygen. In contrast, AEs in Groups A and B were fewer and generally milder; for example, anxiety in the Post-Anesthesia Care Unit (PACU) was noted in 2.3% and 4.7% of patients, respectively, and was categorized as mild and procedural/other in origin. No severe AEs were reported in any group.

**Table 4:** Summary of adverse events by group, severity, and causality.

Adverse Event	Group A (*n* = 86)	Group B (*n* = 85)	Group C (*n* = 85)	Severity	Causality
Anxiety (PACU)	2 (2.3%)	4 (4.7%)	3 (3.5%)	Mild	Procedural/Other
Nausea/Vomiting	0 (0%)	0 (0%)	12 (14.1%)	Moderate	Drug-related
Increased Secretion	0 (0%)	0 (0%)	9 (10.6%)	Mild	Drug-related
Preparatory Depression	0 (0%)	0 (0%)	7 (8.2%)	Moderate	Drug-related
Hypoxemia (SpO_2_ < 95%)	0 (0%)	0 (0%)	3 (3.5%)	Moderate	Drug-related
Other (e.g., agitation)	0 (0%)	0 (0%)	1 (1.2%)	Mild	Procedural/Other
Total	2 (2.3%)	4 (4.7%)	35 (41.2%)^a,b^	‒	‒

Data presented as number (percentage within group). Statistical analyses: χ² test for between-group comparisons. Total events: *p* < 0.01 (Group C vs. A^a^ and vs. B)^b^. Note: Percentages are within-group; totals may exceed individual events due to multiples per patient in Group C. No severe events occurred.

## Discussion

Preoperative anxiety is a prevalent issue in preschool children, contributing to hemodynamic instability during anesthetic induction and adversely affecting postoperative recovery, sometimes resulting in serious complications.^10^^,^^11^ To mitigate preoperative anxiety, facilitate smooth parent-child separation, minimize fear during face mask adaptation, and promote cooperation during peripheral venous cannulation, various pharmacological interventions have become routine.^12^^,^^13^ Common agents include midazolam, ketamine, and dexmedetomidine.^14^^,^^15^ Evidence suggests that intranasal dexmedetomidine is more effective and associated with fewer side effects compared to oral midazolam, the most widely used premedication in pediatric patients.^16^ However, poor compliance with intranasal administration in children favors oral administration, particularly of sweet-tasting medications, due to its greater acceptance.^17^^,^^18^ Oral midazolam remains the most frequently used pediatric premedication because of its ease of administration, rapid onset, short duration of action, and low cost.^19^ Nevertheless, oral midazolam alone may cause agitation, hallucinations, behavioral disturbances, and delayed recovery.^20^ Furthermore, it only provides minimal to moderate sedation, which is often insufficient for peripheral venous cannulation.^21^ Esketamine, the R-enantiomer of ketamine, is a Novel N-Methyl-d-Aspartate (NMDA) receptor antagonist that induces sedation by blocking NMDA receptors and analgesia by activating opioid receptors.^22^ It offers rapid onset, sedation, analgesia, minimal psychotropic side effects, reduced salivation, and rapid recovery, making it a preferable alternative to ketamine in clinical settings. However, its oral bioavailability is only 8%‒11%,^23^ restricting its use as a single oral agent. An ideal preanesthetic medication should deliver consistent, predictable outcomes, exhibit good patient tolerability, and present minimal side effects. One potential approach to achieving this is through the synergistic use of multiple drugs, which can enhance sedative effects while minimizing adverse events by allowing for reduced dosages of each individual agent. Previous studies have demonstrated that the combination of oral midazolam and esketamine achieves more satisfactory pre-anesthetic sedation and facilitates peripheral venous access without prolonging recovery or increasing adverse events.^5^^,^^24^ Nonetheless, the optimal dosage ratio for this combination remains underexplored.

To determine the optimal dosage ratio, the authors selected doses based on the ED95 values for oral midazolam (0.8254 mg/kg) and esketamine (8.2125 mg/kg), as described in the Methods section. These doses were chosen to systematically evaluate the synergistic effects of varying proportions of midazolam and esketamine, ensuring that the total effective dose remained consistent relative to their ED95 values. This approach allowed us to explore the balance between efficacy and safety across different ratios. The ratio for combining oral midazolam and ketamine varied across previous studies. For instance, midazolam dosages ranged from 0.2 mg/kg to 0.5 mg/kg, while ketamine dosages spanned 1‒5 mg/kg.^5^ These inconsistencies hinder the comparison of sedative effects across studies. To identify the optimal combination for pediatric preoperative sedation, the authors initially determined the ED95 for each drug. Using these ED95 values as a reference, various drug ratios were selected for combination. In previous research, the ED95 for oral midazolam was 0.8254 mg/kg,^7^ while the ED95 for oral esketamine was 8.2125 mg/kg (unpublished data), as detailed in the Methods section. In this study, participants were divided into three groups based on these ED95 values: Group A (75% ED95 midazolam, 0.62 mg/kg, and 25% ED95 esketamine, 2.05 mg/kg), Group B (50% ED95 midazolam, 0.41 mg/kg, and 50% ED95 esketamine, 4.1 mg/kg), and Group C (25% ED95 midazolam, 0.21 mg/kg, and 75% ED95 esketamine, 6.16 mg/kg). The present results showed that Group B had the highest sedation success rate among the three groups, with a lower incidence of adverse events during both sedation and recovery periods. Additionally, Group B achieved a higher rate of deep sedation than Group A, but a lower rate than Group C, which experienced the highest incidence of adverse events among the three groups. Overall, the combination of 0.41 mg/kg oral midazolam and 4.1 mg/kg oral esketamine (Group B) was the most effective, achieving the highest sedation success rate with the fewest adverse events. While the absolute difference in sedation success between Groups A and B was statistically significant, its clinical relevance is noteworthy: this improvement translates to approximately 1 in 5 additional children achieving successful sedation without rescue interventions, facilitating smoother parent-child separation and mask acceptance. In perioperative management, this could reduce procedural delays by minimizing the 18.3% lower rescue sedation rate in Group B (vs. A), potentially streamlining operating room efficiency and decreasing overall anesthetic requirements. Furthermore, the enhanced success correlated with higher parental satisfaction scores in Group B (mean 4.2 vs. 3.5 in A, *p* = 0.003), alleviating family distress and fostering a more positive perioperative experience, which may mitigate long-term behavioral sequelae associated with untreated anxiety.^10^^,^^11^

Gender-based analysis did not reveal significant differences in sedation success rates or adverse event rates within each group, suggesting that the efficacy and safety of the midazolam and esketamine combinations are consistent across males and females. This finding supports the generalizability of these results to both genders, although further studies with larger sample sizes may be needed to confirm these observations.

The underlying reason why Group B exhibited the best sedative effect with the fewest adverse events remains unclear, but the authors speculate that the optimized balance between midazolam and esketamine in Group B played a critical role. Considering the oral bioavailability of esketamine, which is approximately 8%‒11%,^23^^,^^25^ the estimated absorbed doses of esketamine in Groups A, B, and C were 0.164‒0.226 mg/kg, 0.328‒0.451 mg/kg, and 0.493‒0.678 mg/kg, respectively. Previous studies suggest that the sub-anesthetic dose of ketamine is 0.3‒0.5 mg/kg.^26^ Given that esketamine is twice as potent as ketamine,^27^ the sub-anesthetic dose range for esketamine is believed to be 0.15‒0.25 mg/kg intravenously.^28^ The absorbed esketamine dose in Group A falls within this sub-anesthetic range, which is typically associated with minimal sedation and analgesia without loss of consciousness. Therefore, the sedative effect in Group A was likely driven primarily by the 0.62 mg/kg dose of oral midazolam, which exceeds the typical oral dose for mild to moderate sedation in children (0.25‒0.5 mg/kg).^21^ As a result, most children in Group A demonstrated minimal to moderate sedation, with only 2.3% reaching deep sedation, consistent with sedation levels reported in previous studies for oral midazolam alone.^21^ In contrast, the absorbed esketamine dose in group C approached the anesthetic induction range, while the oral midazolam dose (0.21 mg/kg) was below the minimum effective dose for sedation.^21^ Thus, sedation in Group C was primarily driven by esketamine. Due to the high esketamine dose, which reached anesthetic induction levels, Group C had the highest incidence of deep sedation and adverse events. In Group B, the effective esketamine dose fell between the sub-anesthetic and anesthetic induction levels. The combination of midazolam and esketamine, with midazolam providing anxiolysis and sedation and esketamine enhancing these effects synergistically without increasing side effects,^29^ produced significantly better outcomes compared to Group A. These findings align with previous research examining the oral combination of midazolam and ketamine.^24^^,^^29^, ^30^, ^31^

Parental satisfaction scores were significantly lower in Group A compared to Groups B and C, directly correlating with the lower sedation success rate observed in Group A. This finding is further supported by the observation that all parents reporting dissatisfaction had children who experienced sedation failure.

The incidence of adverse events was significantly higher in Group C compared to Groups A and B. Adverse events in Group C primarily included increased secretions, nausea, vomiting, and oxygen saturation levels below 95% during the sedation phase, as well as nausea, vomiting, and agitation during recovery. These adverse effects are likely attributable to the higher esketamine dosage in Group C, as esketamine-related side effects such as increased secretions, nausea, vomiting, and agitation are known to be dose-dependent.^32^ As detailed in Table 4, the majority of these were classified as moderate and drug-related, underscoring the dose-dependent risk profile, whereas events in Groups A and B were milder and less frequently attributed to the study drugs. During the sedation phase, three patients in Group C experienced oxygen saturation levels below 95%, which were promptly restored to 100% with nasal cannula oxygenation. This observation contradicts the prevailing belief that esketamine has minimal respiratory depressant effects.^27^ Two possible explanations are: 1) The high esketamine dosage in Group C led to increased secretions and other side effects, possibly causing mild upper airway obstruction and decreased oxygen saturation; and 2) All three patients with reduced oxygen saturation exhibited a modified Ramsay sedation score of 6c or higher (deep sedation) during separation. The proportion of patients reaching deep sedation was significantly higher in Group C (30.6%) compared to Groups B (11.8%) and A (2.3%), suggesting that the risk of respiratory depression increases with deeper sedation levels.

This study has several limitations. Firstly, as a single-center study with a relatively small sample size, it may be necessary to conduct larger-scale studies to further determine the optimal combination and dosage of the oral medications, as well as their efficacy and safety. Secondly, the ED95 value for oral esketamine relied on unpublished pilot data from the present group, which, although internally validated in a similar pediatric population, has not been subjected to external peer review; this use of an unpublished pharmacodynamic reference may introduce potential bias in dose selection and further limit the generalizability of these findings. Future multicenter studies with peer-reviewed ED95 determinations are warranted to confirm these results. Thirdly, the narrow age range (1‒6 years) restricts applicability to broader pediatric populations, such as infants or older children, where pharmacokinetic and behavioral responses may differ. Fourthly, the study lacked long-term follow-up to assess potential behavioral effects, such as emergence of delirium or postoperative maladaptive behaviors, which are known risks associated with preoperative anxiety and sedation. ^10^^,^^13^ Fifthly, the gender-based subgroup analysis, while appropriately included, may have been underpowered due to small subgroup sizes, potentially leading to a Type II error. The non-significant gender differences observed might reflect limited statistical power rather than a true absence of effect, warranting caution in interpreting these preliminary findings. Finally, although Group B demonstrated the best results among the three groups, the sedation success rate did not exceed 90%. Future studies will investigate alternative drug combinations, optimize dosage regimens, evaluate long-term outcomes, and consider patient-specific factors. By conducting these studies, the authors can gain a more comprehensive understanding of the optimal premedication strategies for pediatric patients undergoing surgery and improve the overall patient experience.

## Conclusion

The combination of 0.41 mg/kg oral midazolam and 4.1 mg/kg oral esketamine was found to be a safe and effective regimen for alleviating preoperative anxiety in pediatric patients undergoing elective surgery, with a low incidence of adverse events such as nausea, vomiting, and sedation. Clinically, this regimen's superior success rate offers meaningful advantages in routine practice, including reduced rescue needs and improved parental perceptions, warranting consideration for adoption in pediatric premedication protocols.

## Data availability

The datasets generated and analyzed during the current study are available from the corresponding author on reasonable request.

## Authors’ contributions

Conceptualization: Prof. Siyuan Li, Rongliang Xue and Hongfei Xiong. Project administration: Prof. Siyuan Li, Rongliang Xue and Hongfei Xiong. Funding management: Prof. Siyuan Li and Hongfei Xiong. Data curation: Drs. Guangbo Liu, Yunyun Zhang, Ziwen Wei, Wenwen Liang, Xuemei Zhao and Gang Xiao. Software: Mrs. Jing Liu and Drs. Yingxue Xu. Investigation: Drs. Guangbo Liu, Yunyun Zhang, Ziwen Wei, Wenwen Liang, Xuemei Zhao and Gang Xiao. Writing-original draft: Prof. Hongfei Xiong. Writing-review & editing: Prof. Siyuan Li and Rongliang Xue.

## Funding

This study was supported by the Open Project Funding of Clinical Research Center of Shaanxi Province for Dental and Maxillofacial Diseases, College of Stomatology, Xi’an Jiaotong University (2022YHJB01); Projects of Xi’an International Medical Center Hospital, (2022MS14 and 2023MS01); New Clinical Technology Project of Xi’an International Medical Center Hospital (20,211,205, 20,211,203 & 202,417).

## Declaration of competing interest

The authors declare no conflicts of interest.
